# Calibration and Validation of the Youth Activity Profile as a Physical Activity and Sedentary Behaviour Surveillance Tool for English Youth

**DOI:** 10.3390/ijerph16193711

**Published:** 2019-10-02

**Authors:** Stuart J. Fairclough, Danielle L. Christian, Pedro F. Saint-Maurice, Paul R. Hibbing, Robert J. Noonan, Greg J. Welk, Philip M. Dixon, Lynne M. Boddy

**Affiliations:** 1 Movement Behaviours, Health and Wellbeing Research Group, Department of Sport and Physical Activity, Edge Hill University, St Helens Road, Ormskirk L39 4QP, UK; 2 Faculty of Health and Wellbeing, University of Central Lancashire, Preston PR1 2HE, UK; dchristian@uclan.ac.uk; 3 National Cancer Institute, 9609 Medical Center Drive, Rockville, MD 20850, USA; pedro.saintmaurice@nih.gov; 4 Department of Kinesiology, Recreation, and Sport Studies, University of Tennessee, Knoxville, TN 37996, USA; phibbing@vols.utk.edu; 5 Appetite and Obesity Research Group, Department of Psychological Sciences, University of Liverpool, Bedford Street South, Liverpool L69 7ZA, UK; r.noonan@liverpool.ac.uk; 6 Department of Kinesiology, Iowa State University, Ames, IA 50013, USA; gwelk@iastate.edu; 7 Department of Statistics, Iowa State University, Ames, IA 50013, USA; pdixon@iastate.edu; 8 Physical Activity Exchange, School of Sport and Exercise Sciences, Liverpool John Moores University, 5 Primrose Hill, Liverpool L3 2EX, UK; l.m.boddy@ljmu.ac.uk

**Keywords:** calibration, validation, physical activity, self-report, accelerometer, sedentary behaviour, children, adolescents, measurement

## Abstract

Self-reported youth physical activity (PA) is typically overestimated. We aimed to calibrate and validate a self-report tool among English youth. Four-hundred-and-two participants (aged 9–16 years; 212 boys) wore SenseWear Armband Mini devices (SWA) for eight days and completed the self-report Youth Activity Profile (YAP) on the eighth day. Calibration algorithms for temporally matched segments were generated from the YAP data using quantile regression. The algorithms were applied in an independent cross-validation sample, and student- and school-level agreement were assessed. The utility of the YAP algorithms to assess compliance to PA guidelines was also examined. The school-level bias for the YAP estimates of in-school, out-of-school, and weekend moderate-to-vigorous PA (MVPA) were 17.2 (34.4), 31.6 (14.0), and −4.9 (3.6) min·week^−1^, respectively. Out-of-school sedentary behaviour (SB) was over-predicted by 109.2 (11.8) min·week^−1^. Predicted YAP values were within 15%–20% equivalence of the SWA estimates. The classification accuracy of the YAP MVPA estimates for compliance to 60 min·day^−1^ and 30 min·school-day^−1^ MVPA recommendations were 91%/37% and 89%/57% sensitivity/specificity, respectively. The YAP generated robust school-level estimates of MVPA and SB and has potential for surveillance to monitor compliance with PA guidelines. The accuracy of the YAP may be further improved through research with more representative UK samples to enhance the calibration process and to refine the resultant algorithms.

## 1. Introduction

Physical inactivity and sedentary behaviour (SB) are increasingly prevalent among children and young people [1] and are associated with undesirable health and wellbeing outcomes [2,3]. To further understand physical activity (PA) and SB in youth, it is critical to develop more effective ways to assess these complex behaviours. Accelerometry-based devices have been shown to provide reasonable estimates of both PA [4,5] and SB [6,7] and are widely used in various research applications. Accelerometers rank first in validity among field-based measures of PA/SB and, for that reason, their use in large-scale studies is becoming more common. However, accelerometers are often inaccessible outside of well-funded research studies [8,9,10] or are only used with modestly sized samples due to limited device availability to research teams [11,12,13]. Moreover, accelerometers are not routinely used by PA practitioners, PA and health promotion organisations, or schools, because they are costly and labour-intensive [14] and often require high levels of data processing expertise. Hence, the technological improvements of accelerometers and development of built-in algorithms to assess PA and SB do not translate in direct benefits to health and education professionals, which limits the application of these devices beyond the research community. Although recent analytical advances demonstrate the potential of accelerometers to classify free-living PA states (i.e., inactivity, light PA, moderate PA etc.) [15] and postures (e.g., sitting, standing, walking) [16], on their own, accelerometers are limited in determining PA domains and locations where PA take place, which are often integral to understanding the context underpinning them [17].

Self-report questionnaires of PA and SB overcome some of the limitations of accelerometry, by providing important information about context and setting [18]. Moreover, self-report questionnaires reduce participant burden, and are more affordable than accelerometers when used at scale [14]. Recent systematic reviews identified 89 [19] and 46 [20] different self-report questionnaires to assess youth PA and SB, respectively. The methodological quality of these studies was generally low (e.g., flawed design/methods, insufficient detail about comparator instruments, lack of *a priori* hypotheses, etc.) and none of the questionnaires demonstrated acceptable reliability and validity, with content validity noted as being particularly weak [19,20]. A variety of surveys are used in school and public health surveillance to capture information about PA and SB patterns in youth. In England, no youth PA and SB questionnaire exists that is calibrated to produce acceptable PA and SB estimates, and which is designed to be used easily across different levels of expertise. The Health Survey for England asks youth to recall the frequency and duration of PA over the last seven days to capture in-school and out-of-school sports/activities. However, when compared to accelerometry, this survey under and overestimates moderate-to-vigorous PA (MVPA) in younger and older children, respectively [21]. The recently introduced Sport England Active Lives Survey (ALS) for Children and Young People includes a 7-day recall list of in- and out-of-school activities, when children did these, and for how long [22], but the validity of this instrument has not been established. Both questionnaires estimate PA based on participants’ raw scores and are therefore subject to un-addressed systematic and random error, which are likely to result in estimations of PA that lack equivalence with device-based estimates. Additionally, the ALS does not assess SB. Thus, there is a need for a more accurate self-report methodology to assess both PA and SB in youth that is suitable for youth in England and the wider UK.

Compared to accelerometers, there have been fewer efforts to improve the validity of self-report questionnaires to provide estimates of time spent in PA and SB. One approach is the regression-based calibration of self-report data against accelerometer-derived PA and SB estimates. This method has shown promise in estimating whole-day [23] and context-specific estimates of youth PA [24]. From these studies, Saint-Maurice and Welk developed the Youth Activity Profile (YAP) and demonstrated that coded self-report responses from US youth could be calibrated to provide more accurate estimates of school-level MVPA and SB than self-reported estimates alone [25]. The differences between calibrated YAP estimates and MVPA using SenseWear Armbands (SWA) during school, out-of-school, and at the weekend were −15.6, 3.4, and −21.7 min per week, respectively. Furthermore, calibrated YAP estimates of out-of-school SB time underpredicted SWA estimates by just 49.7 min per week [25]. In a subsequent study, school-level YAP MVPA and SB estimates were within 10%–20% of values obtained from ActiGraph GT3X+ accelerometers [11]. The YAP has been administered by school teachers without any specialist PA/SB measurement knowledge in over 1000 US schools through the National Football League PLAY 60 FITNESSGRAM Partnership Project [26], which demonstrates its utility as a cost-effective population-level PA and SB measurement tool. These findings highlight the value in developing low-cost, standardised, and scalable self-report questionnaires and associated analytical techniques that can produce estimates of PA and SB that are equivalent to those produced by accelerometers [27]. Saint-Maurice and Welk (2015) advocated the testing and potential refinement of the YAP algorithms on independent samples beyond their original study [25]. Reflecting this recommendation, this study aimed to (1) assess the predictive accuracy of applying US-generated YAP calibration algorithms for PA and SB in a sample of English youth, (2) develop and validate English-specific YAP calibration algorithms, and (3) examine their potential surveillance utility to assess compliance to PA guidelines.

## 2. Materials and Methods

### 2.1. Participants and Settings

Eleven schools (five primary and six secondary) from northwest England were informed about the study. Nine of the 11 schools (four primary and five secondary) were recruited and students from randomly selected classes in Year (i.e., Grade) 5 (primary school stage; 10.2 ± 0.3 years), Year 8 (secondary school stage; 13.2 ± 0.3 years) and Year 10 (15.2 ± 0.3 years) were invited to participate (*N* = 409; 212 boys). Informed parental consent and child assent were obtained from 402 participants (209 boys; 98% recruitment rate) who were each assigned to a unique identification code. Each school received a £300 financial incentive for participating, and each participant received a £10 shopping voucher following completion of data collection, which took place between March and July 2017. The study received ethical approval from the Liverpool John Moores University Research Ethics Committee (#14/SPS/012).

### 2.2. Measures

#### 2.2.1. Anthropometric Measures

Height was measured to the nearest 0.1 cm using a portable stadiometer (Seca 213 height measure, Seca UK, Birmingham, UK). Body mass was measured to the nearest 0.1 kg using digital scales (Seca 877 digital scales, Seca UK, Birmingham, UK). Waist circumference was measured to the nearest 0.1 cm using a non-elastic measuring tape, which was positioned around the mid-section of the waist, over the participants’ school shirts. All anthropometric measures were administered and recorded by pairs of trained researchers in accordance with standardised procedures [28]. Height and weight data were converted to body mass index (BMI), which were subsequently used to categorise participants into weight status classifications using International Obesity Task Force BMI cut-points (11).

#### 2.2.2. Socioeconomic Status

Socioeconomic status (SES) was assessed using the 2015 English Indices of Multiple Deprivation (IMD) raw scores [29], which were derived from individual participants’ postcode entries. IMD scores range from 0.5 to 92.6 and are composed of seven domains of deprivation (income, employment, health, education, access to services, living environment and crime), with higher aggregated scores representing higher degrees of deprivation.

#### 2.2.3. Device-Measured Physical Activity and Sedentary Time

PA was measured over eight days using a SenseWear Armband Mini (SWA) (Bodymedia, Inc., Pittsburgh, PA, USA). The SWA is a multi-sensor device that detects and records movement and physiological responses at 60-second epochs (default setting) to provide accurate estimates of energy expenditure. An earlier model (Sensewear Armband Pro) was used in the original US YAP calibration study [25]. A key advantage of the SWA is that its heat flux and temperature sensors automatically detect non-wear time, rather than rely on algorithms based solely on accelerations (e.g., strings of zero counts) [30]. The SWA uses proprietary algorithms to estimate energy expenditure. In this study, algorithm version 5.2 was used. This algorithm is validated for use with children and adolescents and has increased accuracy over the earlier versions [31,32,33]. However, like other devices, measurement error is evident in the SWA, which is more accurate at the school level than at the individual level [31,32,33]. Notwithstanding these limitations, the SWA may be more preferable for estimating energy expenditure in youth than accelerometer-only devices such as the ActiGraph, because of its superior accuracy during cycling which is a common free-living activity in these age groups [31]. Participants were instructed to wear the SWA on the back of the upper arm in direct contact with the skin. They were asked to only remove the device for water-based activities, such as bathing or swimming.

#### 2.2.4. Self-Reported Physical Activity and Sedentary Time

Self-reported PA and SB data were collected using the YAP [25]. The YAP is an online 15-item questionnaire comprised of three sections (school day, out-of-school, and SB), with five questions per section. Participants are asked to recall their PA and SB over the past 7 days during context-specific time segments. For example, the school day questions ask on how many days participants undertook active travel to and from school, and their activity levels during break time, lunch time, and PE. The out-of-school segment refers to activity levels before school, immediately after school, evening, and across both Saturday and Sunday. The SB section asks about time spent watching TV, playing video games, using a mobile phone, a computer/tablet, and overall SB. All questions are structured using a 5-point Likert scale (e.g., for active travel to school, a score of 1 indicates 0 days per week of active travel, whereas a score of 5 indicates 4–5 days per week). The questions referring to break time, lunch time, and PE also include the option for participants to indicate that these PA opportunities did not occur during the previous week. In such instances, a score of 0 is assigned.

Prior to the study commencing, the YAP was minimally amended by the research team to make the clarity, language, and terminology more appropriate for English youth (e.g., the word ‘recess’ was replaced with ‘break time’, ‘cell phone’ was replaced with ‘mobile phone’, etc.). Through this process, the fundamental content and meaning of the YAP questions were unaltered. Differences between the two YAP versions are highlighted in Document S1. Participants completed the YAP using desktop PCs or iPads in classrooms eight days after receiving the SWA. This was to ensure the seven-day recall of the YAP temporally matched the collected SWA data. All participants received the same instructions on how to complete the YAP from a prepared script. Research staff were on hand throughout to assist with any further questions. On completion of the YAP, researchers used recall ‘probing’ questions as a quality assurance mechanism to improve the accuracy of responses [25]. These probes were specifically developed for the YAP calibration and are not part of the YAP, nor are recommended for field applications when using the tool [25]. The English YAP version used in this study is provided as Document S2.

### 2.3. Study Design

The study followed a similar protocol as that detailed in the original US YAP calibration study [25]. Data were collected on a two-week cycle which consisted of two data collection visits to the schools. At the first visit, participants were provided with instructions on how to wear the SWA devices, which were distributed during this session. Anthropometric measures were also obtained. On the second data collection visit (8 days after the first visit), SWA devices were collected and the YAP was administered. Individual students’ home postcodes, ethnicity, and sex were obtained via schools’ information management systems. Schools also provided details of the previous week’s school timetable schedule, which included days and times for school start and end, recess, lunchtime, and physical education (PE) lessons.

### 2.4. Data Processing

#### 2.4.1. Predictive Ability of US Algorithms with an English Sample (Aim 1)

Data were processed using an identical data processing routine to that used in the original US YAP calibration study [25]. SWA data were downloaded using the BodyMedia SenseWear Professional Software v8.0. The SenseWear software automatically detected non-wear time and classified the data into PA or SB on a per minute basis, which is the default setting. Epochs spent in PA ≥4.0 metabolic equivalents (METs) were classified as MVPA, and epochs spent in activities ≤2.0 METs were classified as SB [34,35]. SWA MVPA and SB data were then temporally allocated to specific time segments which corresponded to the time segments integrated into the YAP questions (Table 1; e.g., the SWA data between 18:00 and 22:00 was classified as ‘Evening’). The process of segmentation was conducted in R [36], using code specifically written for this purpose. This generated the number of minutes in MVPA and SB for each segment (e.g., break time, lunch) from the SWA.

The structure and questions of the YAP were designed to temporally link the SWA data to the recall responses. The first 10 questions captured a discrete time segment in which there were specific opportunities to be physically active (Table 1). Previous research has shown that the YAP segments capture 94.6% of the total MVPA that occurs throughout the day [25]. Procedures for scoring the YAP values and converting them to estimates of MVPA and SB are presented in Document S3 and are briefly summarised here. After the YAP data were checked and cleaned, the segment-specific US YAP algorithms [25] were applied to the raw scores for questions 1 to 10 to generate percentage time in MVPA per question, and to the aggregated out-of-school SB score to generate percentage time in out-of-school SB. These percentage values were then multiplied by the duration of each segment (e.g., break time), which was determined by the YAP protocol and school-specific schedules (Table 1), to give the average minutes in MVPA per segment per day. These values were subsequently multiplied by the number of days per week per segment (e.g., 5 days for break time), which resulted in estimates of the average number of minutes in MVPA per week. The same procedure was used with the percentage out-of-school SB values, to produce absolute estimates of daily and weekly SB. The YAP data were then aggregated to reflect estimates of MVPA in-school (questions 1–5), out-of-school (questions 6–8), and at the weekend (questions 9–10), and SB out-of-school (questions 11–15). These data were temporally matched with the corresponding MVPA and SB estimates from the SWA to assess the predictive ability of the US YAP algorithms in an English population. This data segmentation process was conducted at the individual participant level according to the school day schedules provided by each school.

A key aim of our study was to validate a previously developed approach to calibration [11,25]. Hence, it was essential to replicate the methods including the same compliance criteria and representation of ‘typical’ activity. Compliance criteria were that the SWA had to be worn for ≥70% of the corresponding segment durations (e.g., break time) on at least three days [11]. Additionally, the number of valid days required for SWA wear during PE classes and weekend days was set to one. We are unaware of studies that have conducted parallel examinations for specific segments of the week, and we therefore used a conservative approach requiring at least three sessions (i.e., days) for each segment on the basis that less variability in three segment specific replicates would be expected when compared to three full days. Previous studies of segment-specific PA have also used at least three weekdays and one weekend day as a representation of the week [37,38,39], and these criteria exceed those suggested for reliable PA estimates from accelerometers [40]. Following segmentation, individual records were screened for segment-specific compliance to ensure quality and representativeness of the data. Participants with incomplete YAP data were also removed and not considered for further analysis. From the initial sample of 402 participants, after compliance checks, 331 (82%; 170 boys) were included in the final analytical sample.

Agreement between the US YAP algorithm-predicted in-school, out-of-school, and weekend MVPA min·week^−1^ and out-of-school SB min·week^−1^, and corresponding estimates from the SWA were examined at the student level (i.e., variability in agreement between each individual participant’s YAP and SWA estimates) and school level (i.e., variability in agreement between aggregated YAP and SWA estimates for each school). The validity of each of the time segments was analysed at the student level using correlations and the mean absolute percentage error (MAPE), while school-level agreement was determined using the overall mean error or bias between observed and predicted values.

#### 2.4.2. Generation of English-Specific YAP Algorithms (Aim 2)

*Calibration*. The analytical sample data (*n* = 331) were randomly allocated by school (stratified by school stage) into calibration and cross-validation data sets. The calibration data (6 schools; 3 primary and 3 secondary) were used to generate the YAP prediction equations for in-school, out-of-school, and weekend MVPA and out-of-school SB. SWA and English YAP data were processed identically to the previous US YAP studies [11,25]. During calibration, daily percent time in SWA-derived MVPA and SB were treated as the dependent variables. School stage (primary or secondary school), sex, and the corresponding YAP segment composite scores were the independent variables. Quantile regression models [41] were fit separately for each time segment (in-school, out-of-school, and weekend) for MVPA and SB (out-of-school only). Preliminary evaluations considered different combinations of variables and their two-way interactions. Some models did not have unique solutions, and these were not considered further. The accuracy of the final models was examined using root mean square error (RMSE). Calibration analyses were completed in R using the quantreg [42], tidyverse [43], and modelr packages [44].

*Cross-validation.* Data from the remaining three schools (1 primary and 2 secondary) were used to independently assess the prediction accuracy of the English YAP algorithms from the calibration phase. Agreement was investigated by converting the YAP composite segment scores into weekly minutes of MVPA or SB using the algorithms developed in the calibration analyses. Student-level agreement for each YAP segment was determined using correlations and MAPE. Bias was calculated to explore school-level agreement. Equivalence testing was also applied with the cross-validation sample to examine whether 90% confidence intervals (CI) for YAP-predicted minutes of MVPA/SB were within a 10% range (equivalence zone) of estimates from the SWA [45]. Where there was no evidence of equivalence at 10%, the equivalence zone was increased by 5% until equivalence was reached (i.e., 15%, 20%, etc.).

#### 2.4.3. Potential Surveillance Utility of the English YAP Algorithms (Aim 3)

To examine the potential of the English YAP algorithms for PA surveillance, the ability of the YAP to identify participants that met PA guidelines was assessed, using the SWA data as the criterion. Using the full analytical sample (*n* = 331), average MVPA min·day^−1^ and average MVPA min·school day^−1^ were computed for YAP-predicted values and SWA values. Binary codes were used to classify participants according to whether they achieved at least an average of 60 min·day^−1^ MVPA, which reflects current PA recommendations in the UK [46] and internationally [47]. The same method was used for an average of 30 min·school day^−1^ MVPA, which reflects recommendations in the UK [48] and US [49] that schools provide opportunities for youth to achieve at least 50% of the daily recommended PA during school time. Agreements between the proportion of participants achieving the respective 60 and 30 min MVPA recommendations according to the YAP and SWA were compared and the classification accuracy of the YAP was evaluated using percent agreement, kappa, sensitivity, and specificity.

## 3. Results

### 3.1. Descriptive Statistics

The descriptive characteristics of the participants who were included in the analyses are detailed in Table 2. There were no significant differences in age, BMI, and SES between participants included and not included in the analytical sample (*p* > 0.05; i.e., those who did not have complete YAP data and/or who not meet the SWA wear time criteria).

### 3.2. Predictive Ability of US YAP Algorithms with an English Sample (Aim 1)

Correlations between US YAP-predicted and SWA estimates were weak to moderately strong, ranging from r = 0.09 (in-school MVPA) to r = 0.73 (out-of-school SB; Table S4). Agreement was very poor with high MAPE values between 59.0% and 93.6% (Table S4). The US YAP algorithms performed somewhat better at the school level but agreement was still weak (Table 3), with bias ranging from −32.1 (116.8) min·week^−1^ for in-school MVPA to 445.7 (106.4) min·week^−1^ for out-of-school SB.

### 3.3. Generation of English YAP Algorithms (Aim 2)

#### 3.3.1. Calibration

For the calibration analyses, 200 participants had valid YAP and SWA data for at least one of the YAP segments of the week. In the final models, the predictors of MVPA and SB were school level, sex, and the interaction between the segment YAP score and school level (Table 4). RMSE was 12.1%, 9.6%, 8.5%, and 15.3% for in-school, out-of-school, and weekend MVPA, and out-of-school SB, respectively. More detailed summaries of the calibration models and resultant level- and sex-specific algorithms are presented in Document S5.

#### 3.3.2. Cross-Validation

**Figure 1:**
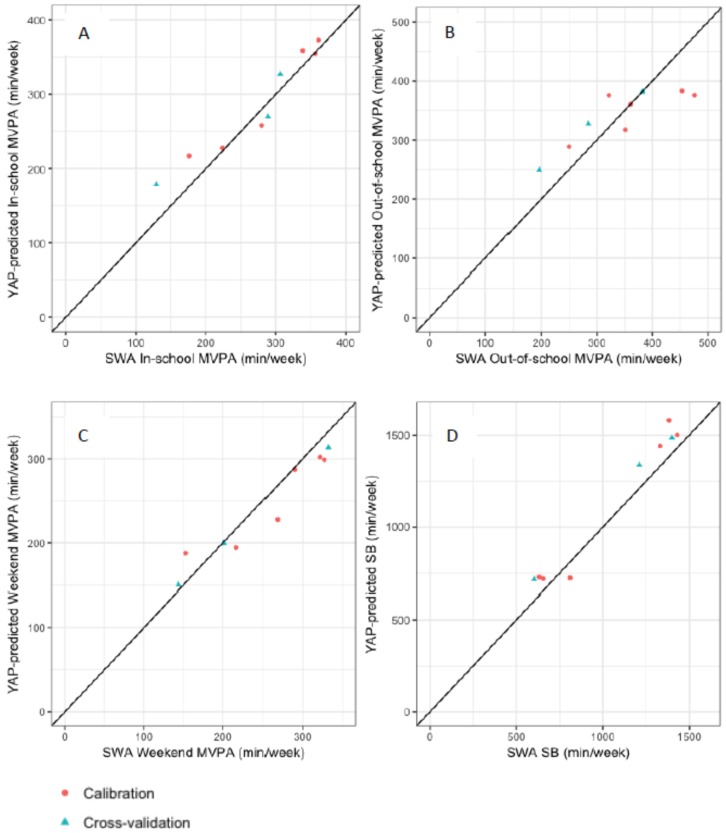
School-level agreement between SWA estimates of MVPA and SB and predicted MVPA and SB minutes using the English YAP prediction algorithms. (**A**) in-school MVPA; (**B**) out-of-school MVPA; (**C**) weekend MVPA; (**D**) out-of-school SB. Each data point represents one school; red dots: the calibration sample schools used for generating the English YAP equations; blue triangles: the cross-validation sample schools used to assess prediction accuracy.

From the three cross-validation schools, there were 129 participants with valid YAP and SWA data for at least one YAP segment of the week. Results are presented separately for each of the four segments. Student-level results are summarised in Table S6. School-level results are presented in Table 5 (min·week^−1^ in MVPA and SB for cross-validation sample), Figure 1 (plots of YAP-predicted and SWA-estimated MVPA and SB for all schools), and Table S7 (percent of segment time in MVPA and SB for cross-validation sample).

*In-school MVPA.* Student-level YAP predicted in-school MVPA min·week^−1^ and SWA-derived MVPA were weakly correlated (r = 0.11), and the MAPE was 70.8%. School-level estimates of MVPA from the YAP and SWA differed by 17.2 (34.4) min·week^−1^ (approximately 3.4 min·day^−1^). The upper and lower limits of the 90% CI for in-school YAP MVPA (90% CI = 241.0, 282.6 min·week^−1^) were within 20% of SWA-estimated MVPA (20% zone = 195.5, 293.3 min·week^−1^) (Table 5).

*Out-of-school MVPA.* The correlation between student-level out-of-school MVPA predicted by the YAP and MVPA from the SWA was r = 0.45 and the MAPE was 83.9%. The YAP over-estimated school-level MVPA compared to SWA estimates by 31.6 (28.3) min·week^−1^ (6.3 min·day^−1^), and predicted and SWA-estimated out-of-school MVPA reached agreement when the equivalence zone was set at 20% (20% zone = 232.5, 348.7 min·week^−1^).

*Weekend MVPA.* Weekend MVPA estimates from the YAP and SWA were moderately correlated (r = 0.52) and the MAPE was very high (199.6%). At the school-level, the YAP under-predicted weekend SWA MVPA by 4.9 (13.2) min·week^−1^ (−2.5 min·day^−1^). The 90% CI for the weekend MVPA YAP values (203.4, 245.8 min·week^−1^) were within the 15% equivalence zone (195.1, 263.9 min·week^−1^).

*Out-of-school SB.* At the student-level, out-of-school time in SB was strongly correlated to SB estimated from the SWA (r = 0.80), and MAPE was 50.6%. School-level YAP-predicted out-of-school time spent in SB was 109.2 (20.5) min·week^−1^ (21.8 min·day^−1^) higher than the SWA estimates. YAP-predicted and SWA-estimated out-of-school SB reached agreement when the equivalence zone was set at 15% (15% zone = 897.9, 1214.9 min·week^−1^).

As the final calibration algorithms were specific to school-level and sex, additional sub-group agreement analyses were performed separately for all primary and secondary schools (Table 6), and for all boys and girls (Table 7). Bias ranged from 12.9 (10.6) min·week^−1^ (in-school MVPA) to 47.2 (91.5) min·week^−1^ for SB in primary schools, and from −4.5 (28.8) min·week^−1^ (weekend MVPA) to 118.4 (50.0) min·week^−1^ (SB) in secondary schools. Primary school YAP-predicted and SWA-estimated in-school and weekend MVPA demonstrated the closest agreement (10% equivalence zone). For secondary school students, weekend MVPA and SB were equivalent in the 15% zone. Between-segment differences in bias were similar for boys and girls. These ranged from −2.5 (51.6) min·week^−1^ (boys) and −3.9 (71.1 min·week^−1^ (girls) for out-of-school MVPA, to 89.7 (78.2) min·week^−1^ (boys) and 61.2 (84.1 min·week^−1^ (girls) for out-of-school SB. Boys’ YAP-predicted and SWA-estimated out-of-school MVPA demonstrated the closest agreement (10% equivalence zone). For girls, in-school and out-of-school MVPA were equivalent in the 15% zone. These additional school-level analyses are also presented in Table S7 with percent of segment time as the outcome.

### 3.4. Classification Accuracy of the YAP (Aim 3)

According to the SWA, 60 min·day^−1^ MVPA was achieved by 81% of the participants. YAP-predicted estimates of daily MVPA indicated that the recommendation was met by 85.8% of participants. Agreement was 80.7% and the kappa value was 0.31 (fair agreement). Sensitivity and specificity were 91% and 37%, respectively. The school day 30 min·day^−1^ MVPA recommendation was achieved by 77.6% and 79.2% of participants, according to SWA and YAP-predicted estimates, respectively. Percent agreement and kappa values were 82.2% and 0.47, respectively (moderate agreement). The classification accuracy was 89% sensitivity and 57% specificity.

## 4. Discussion

This study aimed to examine the predictive accuracy of the US YAP algorithms for MVPA and SB in a sample of English youth, and to calibrate and test the validity and predictive utility of new English YAP algorithms. We found that the US YAP algorithms poorly predicted SWA estimates of MVPA and SB in English youth. School-level predictions of in-school, out-of-school, and weekend MVPA, and out-of-school SB from the English YAP algorithms were promising, and the YAP demonstrated potential as a surveillance tool to identify prevalence of compliance to youth PA guidelines.

### 4.1. Aim 1

There was poor student- and school-level agreement between the US YAP estimates of MVPA and SB and estimates from the SWA. The recommendation of Saint-Maurice and Welk that the US YAP algorithms be tested and refined on independent samples [25] is therefore well founded. Moreover, it reinforces the notion that the content of self-report PA questionnaires is population- and context-specific, and that questionnaires developed and validated in one population cannot automatically be assumed to be suitable for youth elsewhere with different PA contexts and routines [50]. Cross-cultural differences exist relating to individuals as well as the contexts and settings in which they live (e.g., school day schedules, sports practices, home routines, etc.) [51]. Although the development of the US YAP algorithms used the same data processing steps and a similarly aged-sample as in the present study, critically, the algorithms reflected the school schedules and out-of-school routines of the US youth, which differed to those in our English sample. These differences were reflected in the high MAPE values and degree of bias when the US YAP algorithms were applied to the English SWA data.

### 4.2. Aim 2

When the estimates from the English YAP algorithms were compared to those from the SWA, student-level agreement was poor. This is consistent with what has been observed previously for the YAP [11,25], for other self-report instruments [19,20], and also for most calibration equations developed for accelerometers [52]. The high MAPE values exceeded those observed when the YAP was compared with MVPA and SB estimates from 12–17-year-olds wearing wrist-mounted ActiGraph GT3X+ accelerometers [11]. These results may have been influenced by a relatively high number of small observed SWA-derived MVPA and SB estimates, which when combined with moderate absolute error values result in very large MAPE values [53]. The data clearly indicate that the YAP estimates of MVPA and SB lack predictive accuracy at the level of the student, and therefore should not be used to inform or evaluate student-level intervention or PA prescription.

School-level bias between English YAP-predicted MVPA and SB and estimates from the SWA were substantially better than those from the student-level analyses. The degree of bias between in-school YAP and SWA MVPA was comparable with previous YAP validations [11,25]. Further, the YAP-predicted in-school MVPA was equivalent to SWA estimates at 20% equivalence. This is the same as that reported in the original US YAP calibration study using SWA devices [25], but higher than the 15% equivalence observed in a subsequent study using the ActiGraph GT3X+ [11]. The out-of-school MVPA bias of 31.6 min·week^−1^ was much higher than the −3.0 min·week^−1^ [11] and 3.4 min·week^−1^ [25] previously reported for US youth. YAP and SWA out-of-school MVPA were deemed equivalent at 20%, whereas Saint-Maurice and colleagues reported 15% [25] and 10% equivalence [11] in their US studies. Weekend MVPA was predicted to within −4.9 min min·week^−1^ of the SWA estimates, which is substantially less than the −21.7 min·week^−1^ and −17.8 min·week^−1^ observed by Saint-Maurice and Welk [25] and Saint-Maurice et al. [11], respectively. Further, the 15% equivalence between weekend MVPA from the YAP and SWA was superior to the US studies (30% [25] and 20% [11]). Conversely, bias and equivalence for out-of-school SB predicted by the YAP was higher than Saint-Maurice and colleagues reported [11,25].

A key difference between the English and US YAP estimates was over-prediction of in-school and out-of-school MVPA, and SB. The English YAP algorithms were based on the specific school schedules and daily routines of the participants in the calibration sample, although the same data processing methods as the previous YAP studies were used [11,25]. It is established that PA recall methods are subject to various sources of measurement error [54], and in this study the over-predictions may have reflected such factors related to the data collection protocol and the participants themselves. For example, though the research assistants received the same training there may have been variation in how and to what extent they used the probing questions to check participants’ YAP recall accuracy. Moreover, differences in literacy and cognitive understanding of the YAP questions likely varied among the participants, and particularly in the primary school group. Even though the probing questions were employed, variations in how the YAP questions were interpreted would have contributed to measurement and processing errors [54], which would have affected the resultant algorithms. The participants in our study were also relatively active and any expected over-estimations of their PA [55] may have been exacerbated when recalling their MVPA behaviours. YAP-predicted SB was also overestimated but the 15% equivalence was promising. SBs tend to be more stable than active behaviours [56]. However, the YAP overestimated SB by almost 22 min·day^−1^, which may reflect that engagement in SBs, like TV viewing, or gaming occurred sporadically rather than as part of a set structure and routine [57]. As a result, it is likely that recall of the specific SBs included in the YAP was challenging for some participants [58,59].

Agreement between YAP-predicted and SWA-estimated MVPA was greatest for the weekend YAP segment. Weekends often involve greater choices and time for recreational activities, but weekend schedules can also reflect regularly occurring activities such as household chores, caring for siblings, and sports practices, etc. Although not as structured as the school day, weekends can represent familiar and routinised contexts for some youth. Moreover, the YAP uses two of the 15 questions to ask about PA during whole weekend days. Longer recall periods (i.e., a full day) are hypothesised to inhibit accurate recall of PA behaviours [60]. It is, however, possible that only focusing on two specific days, rather than five, reduced recall burden and facilitated more accurate responses, which contributed to the low observed error for weekend MVPA.

### 4.3. Aim 3

The English YAP algorithms demonstrated their utility to evaluate compliance with health-related PA recommendations. Sensitivity values associated with the 60 and 30 min MVPA·day^−1^ recommendations indicated that 91% and 89% of youth who achieved the respective recommended daily MVPA would be correctly identified as ‘active’ based on the English YAP algorithm predicted MVPA. These sensitivity values are superior to those reported in the original US YAP calibration study [25] and reinforce the utility of the English YAP algorithms for identifying youth that meet PA guidelines. Saint-Maurice and Welk also reported specificity values of 69% (60 min MVPA·day^−1^) and 61% (30 min MVPA·school-day^−1^) [25], compared to 37% and 57%, respectively in the present study. This suggests that the English YAP MVPA algorithms were relatively less able to accurately classify youth who did not achieve PA guidelines as ‘inactive’. Hence, further refinement of the YAP MVPA algorithms is needed to improve classification accuracy.

### 4.4. Strengths and Limitations

The calibrated YAP estimates of MVPA and SB have great potential utility for future research and PA promotion, as existing calibrated self-report instruments for English youth are not available. Strengths of the study included (1) the use of a proven, rigorous YAP protocol and methodology; (2) the use of manageable group sizes for data collection, which allowed the use of recall probes to enhance the participants’ recall accuracy; (3) the recording of detailed timetable information from each school to accurately determine each participant’s schedule during the week when they wore the SWA, so as to enhance the degree of temporal precision required for the calibration analyses; (4) the use of an independent sample for the cross-validation analyses, and (5) the choice of the SWA as the device-based measure, which has previously demonstrated superior agreement with criterion measures of free-living energy expenditure than other research-grade and consumer activity monitors [31]. There are also limitations which require consideration. Schools were not selected at random and so a degree of sampling bias in favour of more active participants may have been evident. Data were collected in the spring and summer months which may have reflected the relatively high estimates of MVPA. Therefore, the English YAP algorithms do not account for seasonal variation in the participants’ PA and SB. The YAP content means that it can only be used to predict MVPA and SB during school-term time and not during vacation periods, and all modes of MVPA and SB may not be captured. However, schools in England are in session for around 39 weeks of the year so typical activity would be captured by the YAP. The YAP-predicted MVPA and SB estimates demonstrated good school-level agreement, but like values from all PA measurement tools, they cannot be considered exact values reflecting student-level activity behaviours. Moreover, the calibration algorithms are based on MVPA and SB estimates from the SWA as the field-based criterion measure. Further, MVPA and SB are estimated by the SWA algorithm from energy expenditure calculations adjusted for age, sex, and BMI, which are subsequently converted to epoch-level MET values. Thus, like all PA measurement instruments student-level error will be present in these estimations, which is something we could not control, and which may have attenuated the effects of the analyses [11]. Incorporating measurement error modelling against a criterion measure has been shown to help reduce the effects of measurement error and improve the precision of PA estimates from self-report questionnaires [54]. A true criterion measure of free-living PA and SB requires accurate ground-truth measurement (e.g., wearable cameras) to label activity behaviours [61] (although unsupervised machine learning methods are now emerging, which may remove the need for criterion measures [15]). However, these approaches are yet to be feasible in large samples, and therefore, currently offer limited value for the calibration of self-report questionnaires. Lastly, the SWA uses a default 60-second epoch setting to record data, which may not have fully captured intermittent bouts of higher intensity PA that are characteristic of school-aged youth [62]. However, this monitor has been shown to provide valid estimates of PA in this population [31].

## 5. Conclusions

Poor agreement was observed in MVPA and SB derived from the US YAP algorithms and SWA worn by the English sample. YAP algorithms developed using the English sample data resulted in MVPA and SB estimates that had promising school-level agreement with the lowest error observed for weekend MVPA and out-of-school SB. The YAP has potential as a surveillance tool to monitor compliance with youth PA guidelines, but more refinement is needed to improve its classification accuracy. The school-level YAP estimates of MVPA indicate that the YAP is a promising self-report questionnaire for use with English youth, and potentially with samples from other countries in the UK. The YAP is a cost-effective, easy to implement instrument that can be used at scale and implemented by researchers and practitioners to provide meaningful school-level estimates of MVPA and SB. Further refinement of the YAP algorithms with a more representative UK sample and by employing replicate measurement error modelling procedures to enhance the precision of the calibration algorithms is advocated.

## Figures and Tables

**Table 1:** Time segments used in the Youth Activity Profile (YAP) calibration (adapted from [25]).

Question/Segment	Date	Individualised Time	Start Time *	End Time *
1. Before travel to school	Every day	Yes	60 min before start time for travel to school	Start time for travel to school
2. Travel to school	Every day	Yes	30 min before start time for school	Start time for school
3. Play/Break time	When provided	Yes	Determined by school schedule	Determined by school schedule
4. Physical Education	When provided	Yes	Determined by school schedule	Determined by school schedule
5. Lunch	When provided	Yes	Determined by school schedule	Determined by school schedule
6. Travel from school	Every day	Yes	End time for school	30 min after end time for school
7. After school	Every day	Yes	End time for travel from school	18:00
8. Evening	Every day	No	18:00	22:00
9. Saturday	Saturday	No	07:00	22:00
10. Sunday	Sunday	No	07:00	22:00

* Individualised school Start and End times were obtained from individual schools (e.g., start at 09:00 and end at 15:30).

**Table 2:** Descriptive statistics of complete, calibration, and cross-validation samples. (Mean (SD) unless stated).

Variable	All	Calibration Sample	Cross-Validation Sample
*n*	331	202	129
Sex			
Boys (%)	51.4	59.4	38.8
Girls (%)	48.6	40.6	61.2
Ethnicity			
White British (%)	93.7	94.6	92.2
Other (%)	6.3	5.4	7.8
Age (years)	12.3 (2.1)	12.3 (2.1)	12.2 (2.3)
Height (cm)	154.9 (13.7)	155.0 (14.1)	154.6 (13.2)
Weight (kg)	49.6 (15.4)	50.0 (15.3)	49.0 (15.6)
Body mass index (BMI) (kg∙m^2^)	20.3 (4.3)	20.4 (4.4)	20.1 (4.3)
Weight status			
% Normal weight	71.6	71.3	72.1
% Overweight/Obese	28.4	28.7	27.9
Waist Circumference (cm)	71.0 (10.3)	71.4 (10.3)	70.4 (10.2)
Socioeconomic status (SES; 2015 English Indices of Multiple Deprivation (IMD) score)	19.4 (13.9)	21.9 (15.5)	15.7 (10.3)
SenseWear Armband Mini devices (SWA) wear time (days)	5.8 (1.2)	5.8 (1.2)	5.9 (1.3)
SWA total wear time (min⋅day^−1^)	1014.8 (114.7)	1021.9 (115.2)	1004.5 (113.5)

**Table 3:** School-level agreement between SWA and US YAP predicted moderate-to-vigorous PA (MVPA) and sedentary behaviour (SB) (*n* = 9 schools).

Segment	YAP-Predicted Estimates (min·week^−1^)	SWA Estimates (min·week^−1^)	YAP-SWA Bias (min·week^−1^)
In-school MVPA	241.1 (61.4)	273.3 (81.3)	−32.1 (116.8)
Out-of-school MVPA	235.0 (42.6)	341.8 (90.9)	−106.7 (60.1)
Weekend MVPA	175.4 (49.6)	249.1 (74.0)	−73.7 (36.5)
Out-of-school SB	1496.0 (297.8)	1050.4 (365.8)	445.7 (106.4)

**Table 4:** Regression coefficients (SE) for in-school, out-of-school, and weekend MVPA, and sedentary behaviour YAP segments.

Model	In-school MVPA (*n* = 200)	Out-of-School MVPA (*n* = 196)	Weekend MVPA (*n* = 187)	Sedentary Behaviour (*n* = 196)
Intercept (Primary stage)	**45.51 (9.14)**	**12.75 (5.01)**	**15.17 (5.02)**	**34.92 (12.71)**
Intercept (Secondary stage)	**13.72 (5.58)**	2.61 (3.41)	**8.44 (3.18)**	**48.95 (8.91)**
Sex	**−11.34 (2.47)**	−1.61 (1.95)	**−4.33 (1.56)**	1.61 (2.99)
YAP x Primary level	1.78 (2.33)	2.01 (2.06)	1.13 (1.22)	−0.84 (5.22)
YAP x Secondary level	**7.31 (1.66)**	**4.44 (1.18)**	1.44 (0.90)	**5.65 (2.54)**

Note. Bold type indicates significance (*p* < 0.05).

**Table 5:** Cross-validation sample school-level estimates of MVPA and SB, bias, and equivalence.

Segment	YAP-Predicted Estimates (min·week^−1^)	SWA Estimates (min·week^−1^)	YAP-SWA bias (min·week^−1^)	Equivalence Zone
In-school MVPA	258.7 (74.9)	241.4 (97.6)	17.2 (34.4)	20%
Out-of-school MVPA	319.2 (67.0)	287.6 (93.2)	31.6 (28.3)	20%
Weekend MVPA	220.9 (83.6)	225.8 (96.8)	−4.9 (13.2)	15%
Out-of-school SB	1180.3 (408.6)	1071.1 (416.1)	109.2 (20.5)	15%

**Table 6:** School-level estimates of MVPA and SB, relative bias, and equivalence for primary and secondary stages.

Segment	YAP-Predicted Estimates (min·week^−1^)	SWA Estimates (min·week^−1^)	YAP-SWA Bias (min·week^−1^)	Equivalence Zone
Primary stage				
In-school MVPA	353.4 (19.1)	340.5 (24.8)	12.9 (10.6)	10%
Out-of-school MVPA	379.1 (3.9)	408.4 (70.3)	−29.3 (69.5)	25%
Weekend MVPA	300.1 (10.7)	317.9 (19.2)	−17.8 (10.7)	10%
Out-of-school SB	722.1 (5.5)	674.9 (92.8)	47.2 (91.5)	20%
Secondary stage				
In-school MVPA	230.4 (36.0)	219.5 (68.0)	10.9 (33.1)	20%
Out-of-school MVPA	308.3 (42.2)	288.5 (69.4)	19.8 (36.4)	20%
Weekend MVPA	192.0 (28.0)	196.4 (50.7)	−4.5 (28.8)	15%
Out-of-school SB	1469.1 (89.2)	1350.7 (86.2)	118.4 (50.0)	15%

**Table 7:** School-level estimates of MVPA and SB, relative bias, and equivalence for boys and girls.

Segment	YAP-Predicted Estimates (min·week^−1^)	SWA Estimates (min·week^−1^)	YAP-SWA Bias (min·week^−1^)	Equivalence Zone
Boys				
In-school MVPA	342.1 (63.1)	328.7 (66.2)	13.4 (36.9)	15%
Out-of-school MVPA	366.7 (36.3)	369.2 (66.8)	−2.5 (51.6)	10%
Weekend MVPA	282.4 (56.6)	262.7 (69.0)	19.7 (54.2)	20%
Out-of-school SB	1101.0 (426.9)	1011.3 (404.8)	89.7 (78.2)	20%
Girls				
In-school MVPA	242.4 (67.2)	228.7 (81.6)	13.7 (25.4)	15%
Out-of-school MVPA	328.2 (51.4)	332.2 (109.8)	−3.9 (71.1)	15%
Weekend MVPA	211.7 (56.1)	247.8 (86.9)	−36.1 (36.1)	25%
Out-of-school SB	1093.8 (377.5)	1032.6 (348.6)	61.2 (84.1)	15%

## Supplementary Materials

The following are available online at https://www.mdpi.com/1660-4601/16/19/3711/s1: Document S1. Amendments to original YAP wording.pdf; Document S2. YAP.pdf; Document S3. Scoring and conversion of raw YAP values.pdf; Table S4. Student-level results US YAP vs SWA.pdf; Document S5. English YAP calibration models and equations.pdf; Table S6. Student-level cross-validation results minutes and percent segment times.pdf; Table S7. School-level cross-validation results percent segment time.pdf. Availability of data and material: All data generated or analysed during this study are deposited on the Open Science Framework and can be accessed from https://osf.io/zwy6j/.
