# Supplementary material for: Calibration and Validation of the Youth Activity Profile as a Physical Activity and Sedentary Behaviour Surveillance Tool for English Youth
Source: Int J Environ Res Public Health. 2019 Oct 2;16(19):3711. doi: 10.3390/ijerph16193711 (PMC6801945; doi:10.3390/ijerph16193711)
Supplement: Supplementary file 1 [file ijerph-16-03711-s001.zip › Supplementary file 1. Amendments to original YAP wording.pdf]

Youth Activity Profile

About Me

Before you begin, it is important to get some basic information about your school and about you. Provide your birth date, month and year, your gender and year group.

Gender:	Male	Female				
School Level:	Primary School		High/Secondary School			
Year:	5	6	7	8	9	10	11
Date of birth:						

The Youth Activity Profile will ask you about the time you spend being active (both in school and out of school) and the time you spend being sedentary.

Physical activities are things that involve a lot of walking, running or moving around. Physical activities include biking and dancing, as well as sports or outdoor play that involves a lot of moving around, that make you feel warmer and make your heart beat faster.

Sedentary activities are things you do in your free time while sitting or lying down, such as watching TV, playing video games, computer games, using a phone or tablet. Sedentary activities do NOT include the time you spend sitting while eating or while doing homework.

Most questions will ask you only to think about the <u>last 7 days</u> but a few questions will ask about what you typically do (on a normal week). <u>There are no right or wrong answers so please answer as honestly as you can.</u>

Commented [SF1]: US YAP wording:
School Level: Elementary School Middle School High School
Grade: 3 4 5 6 7 8 9 10 11 12

Commented [SF2]: US YAP wording:
Sedentary activities are things such as watching TV, or playing video games, computer games, or hand-held games that you do in your free time. It does NOT include the time you spend sitting while eating or while doing homework.

Youth Activity Profile
Activity Levels at School

These questions ask about your physical activity at school. This includes PE lessons but you may also be active on your way to school, during playtime/break, or at lunch. **Answer the questions based on your physical activity at school in the last 7 days.**

Answer each question by circling one letter

1. Activity to School: In the last 7 days, how many days did you **walk or bike to school**? *(If you can't remember, try to estimate)*

a. 0 days (never)
b. 1 day
c. 2 days
d. 3 days
e. 4-5 days (mostly every day)

2. Activity during PE Lessons: During **PE or games lessons** in the last 7 days, how often were you running and moving as part of the planned games or activities? *(If you didn't have PE or games, choose "I didn't have PE")*

a. I didn't have PE
b. Almost none of the time
c. A little bit of the time
d. About half the time
e. A lot of the time
f. Almost all of the time

For Primary school pupils **3. Activity during Playtime:** During **playtime** in the last 7 days, how often were you playing sports, walking, running, or playing active games? *(If you didn't have a playtime at school, choose "I didn't have playtime")*

a. I didn't have playtimes
b. Almost none of the time
c. A little bit of the time
d. About half the time
e. A lot of the time
f. Almost all of the time

Commented [SF3]: US YAP wording:
These questions ask about your physical activity at school. This includes physical education but you may also be active on your way to school, during recess, or at lunch. **Answer the questions based on your physical activity at school in the last 7 days.**

Commented [SF4]: US YAP wording:
1. Activity to School: How many days did you **walk or bike to school**? *(If you can't remember, try to estimate)*
* The addition of "in the last 7 days" was made to each question

Commented [SF5]: US YAP wording:
2. Activity during Physical Education Class: During **physical education**, how often were you running and moving as part of the planned games or activities? *(If you didn't have PE, choose "I didn't have physical education")*

Commented [SF6]: US YAP wording:
d. A moderate amount of time

Commented [SF7]: US YAP wording:
3. Activity During Recess: During **recess**, how often were you playing sports, walking, running, or playing active games? *(If you didn't have a break at school, choose "I didn't have recess")*

Commented [SF8]: US YAP wording:
d. A moderate amount of time

For Secondary school pupils **3. Activity During Breaks:** During **breaks** in the last 7 days, how often were you playing sports, walking, running, or playing active games? *(If you didn't have a break at school, choose "I didn't have breaks")*

a. I didn't have breaks
b. Almost none of the time
c. A little bit of the time
d. About half the time
e. A lot of the time
f. Almost all of the time

Commented [SF9]: US YAP wording:
For middle and high school.
3. Activity During Breaks/Study Hall: During **breaks/study hall**, how often were you playing sports, walking, running, or playing active games? *(If you didn't have a break at school, choose "I didn't have breaks/study hall")*

Commented [SF10]: US YAP wording:
d. A moderate amount of time

4. Activity during Lunch: During **lunch breaks** in the last 7 days, how often were you moving around, walking or playing? *(If you didn't have a lunch break at school, choose "I didn't have lunch breaks")*

a. I didn't have lunch breaks
b. Almost none of the time
c. A little bit of the time
d. About half the time
e. A lot of the time
f. Almost all of the time

Commented [SF11]: US YAP wording:
d. A moderate amount of time

5. Activity from School: In the last 7 days how many times did you **walk or bike from school**? *(If you can't remember, try to estimate)*

a. 0 days (never)
b. 1 day
c. 2 days
d. 3 days
e. 4-5 days (mostly every day)

Youth Activity Profile
Activity Levels Out of School

These questions ask about your physical activity out of school. This includes activities before and after school, in the evening and at weekends. **Answer the questions based on your physical activity out of school in the last 7 days**.

6. Activity before School: In the last 7 days, how many times **before school (6:00-8:00 am)** did you do some form of physical activity for at least 10 minutes? *(This includes activity at home NOT walking or biking to school)*

a. 0 days
b. 1 day
c. 2 days
d. 3 days
e. 4 to 5 days

Commented [SF12]: US YAP wording:
6. Activity before School: How many days **before school (6:00-8:00 am)** did you do some form of physical activity for at least 10 minutes? *(This includes activity at home NOT walking or biking to school)*

7. Activity after School: In the last 7 days, how many times **after school (between the end of school and 6:00 pm)** did you do some form of physical activity for at least 10 minutes? (This can include playing with your friends/family, team practices or classes involving physical activity but *NOT walking or biking home from school*)

a. 0 days
b. 1 day
c. 2 days
d. 3 days
e. 4 to 5 days

Commented [SF13]: US YAP wording:
7. Activity after School: How many days **after school (between 3:00 - 6:00 pm)** did you do some form of physical activity for at least 10 minutes? (This can include playing with your friends/family, team practices or classes involving physical activity but *NOT walking or biking home from school*)

8. Activity on Weeknights: In the last 7 days, on how many **school evenings (6:00 - 10:00 pm)** did you do some form of physical activity for at least 10 minutes? (This can include playing with your friends/family, team practices or classes involving physical activity but *NOT walking or biking home from school*)

a. 0 days
b. 1 day
c. 2 days
d. 3 days
e. 4 to 5 days

Commented [SF14]: US YAP wording:
8. Activity on Weeknights: How many **school evenings (6:00 - 10:00 pm)** did you do some form of physical activity for at least 10 minutes? (This can include playing with your friends/family, team practices or classes involving physical activity but *NOT walking or biking home from school*)

9. Activity on Saturday: How much physical activity did you do last **Saturday**? *(This could be for exercise, work/chores, family outings, sports, dance, or play. If you don't remember, try to estimate)*

a. No activity (0 minutes)
b. Small amount of activity (1 to 30 minutes)
c. Small to Moderate amount activity (31 to 60 minutes)
d. Moderate to Large amount of activity (1 to 2 hours)
e. Large amount of activity (more than 2 hours)

10. Activity on Sunday: How much physical activity did you do last **Sunday**? *(This could be for exercise, work/chores, family outings, sports, dance, or play. If you don't remember, try to estimate)*

a. No activity (0 minutes)
b. Small amount of activity (1 to 30 minutes)
c. Small to Moderate amount activity (31 to 60 minutes)
d. Moderate to Large amount of activity (1 to 2 hours)
e. Large amount of activity (more than 2 hours)

Youth Activity Profile

Sedentary Activities

These questions ask about your sedentary activities out of school. Sedentary activity usually involves sitting or lying down and includes things like watching TV, playing video games, and using a mobile phone. **Answer the questions based on your sedentary activities out of school in the last 7 days.**

11. TV Time: In the last 7 days, how much time did you spend **watching TV** outside of school time *(This includes time spent watching TV programmes, movies or sports but NOT time spent playing video games)*

a. I didn't watch TV at all
b. I watched less than 1 hour per day
c. I watched 1 to 2 hours per day
d. I watched 2 to 3 hours per day
e. I watched more than 3 hours per day

12. Video Game Time: In the last 7 days, how much time did you spend **playing video games** outside of school time? *(This includes games on things like Nintendo DS, wii, Xbox, PlayStation, iPod Touch, iPad, or games on your PC and phone)*

a. I didn't play at all
b. I played less than 1 hour per day
c. I played 1 to 2 hours per day
d. I played 2 to3 hours per day
e. I played more than 3 hours per day

13. Computer/tablet Time: In the last 7 days, how much time did you spend using **computers or tablets** outside of school time? *(This doesn't include homework time and playing online video games or computer games, but does include time on things like Instagram and Facebook, as well as time spent surfing the internet, and instant messaging)*

a. I didn't use the computer/tablet at all
b. I used a computer/tablet less than 1 hour per day
c. I used a computer/tablet 1 to 2 hours per day
d. I used a computer/tablet 2 to 3 hours per day
e. I used a computer/tablet more than 3 hours per day

Commented [SF15]: US YAP wording:
Sedentary Habits

Commented [SF16]: US YAP wording:
11. TV Time: How much time did you spend **watching TV** outside of school time *(This includes time spent watching movies or sports but NOT time spent playing video games)*.

Commented [SF17]: US YAP wording:
12. Video Game Time: How much time did you spend **playing video games** outside of school time? *(This includes games on Nintendo DS, wii, Xbox, PlayStation, iTouch, iPad, or games on your phone)*

Commented [SF18]: US YAP wording:
13. Computer Time: How much time did you spend using **computers** outside of school time? *(This doesn't include home work time but includes time on Facebook as well as time spent surfing the internet, instant messaging, playing online video games or computer games)*

Commented [SF19]: US YAP wording:
a. I didn't really use the computer at all
b. I used a computer less than 1 hour per day
c. I used a computer 1 to 2 hours per day
d. I used a computer 2 to3 hours per day
e. I used a computer more than 3 hours per day

14. Phone Time: In the last 7 days, how much time did you spend using your **mobile phone** after school? *(This includes time spent talking, texting, or using things like Instagram and Facebook, but does not include playing games)*.

a. I didn't use a mobile phone
b. I used a phone less than 1 hour per day
c. I used a phone 1 to 2 hours per day
d. I used a phone 2 to 3 hours per day
e. I used a phone more than 3 hours per day

15. Overall Sedentary Habits: Which of the following best describes your **typical** sedentary habits at home? *(Try to think about a typical week and not just last week)*

a. I spent almost none of my free time sitting
b. I spent little time sitting during my free time
c. I spent about half of my free time sitting
d. I spent a lot of time sitting during my free time
e. I spent almost all of my free time sitting

Commented [SF20]: US YAP wording:
14. Phone / Text Time: How much time did you spend using your **cell phone** after school? *(This includes time spent talking or texting)*.
