# Supplementary material for: Calibration and Validation of the Youth Activity Profile as a Physical Activity and Sedentary Behaviour Surveillance Tool for English Youth
Source: Int J Environ Res Public Health. 2019 Oct 2;16(19):3711. doi: 10.3390/ijerph16193711 (PMC6801945; doi:10.3390/ijerph16193711)
Supplement: Supplementary file 1 [file ijerph-16-03711-s001.zip › Supplementary file 4 REVISED. Individual-level results US YAP vs SWA.pdf]

US YAP agreement with SWA results

Individual-level

	YAP (min/week)	SWA (min/week)	YAP-SWA bias (min/week)	%MAPE
In school MVPA n=329	242.7 (95.8)	259.8 (123.3)	-17.1 (149.5)	93.4%
Out school MVPA n=323	228.4 (67.2)	324.8 200.5)	-96.4 (181.8)	59.0%
Weekend MVPA n=306	166.0 (237.4)	237.4 (162.3)	-71.4 (147.3)	93.6%
Out school ST n=303	1548.7 (310.1)	1117.6 (448.6)	431.1 (305.1)	84.5%
