# Supplementary material for: Calibration and Validation of the Youth Activity Profile as a Physical Activity and Sedentary Behaviour Surveillance Tool for English Youth
Source: Int J Environ Res Public Health. 2019 Oct 2;16(19):3711. doi: 10.3390/ijerph16193711 (PMC6801945; doi:10.3390/ijerph16193711)
Supplement: Supplementary file 1 [file ijerph-16-03711-s001.zip › Supplementary file 5. English YAP calibration models & equations.pdf]

English YAP calibration models and equations

In-school MVPA calibration model

n=200	ß	SE	p
Intercept (Primary level)	45.51	9.14	<.001
Intercept (Secondary level)	13.72	5.58	.011
Sex	-11.34	2.47	<.001
YAP x Primary level	1.78	2.33	.45
YAP x Secondary level	7.31	1.66	<.001

Calibration equations

- Primary boys: YAP % MVPA = 45.51 + 1.78 * YAP score
- Primary girls: YAP % MVPA = 45.51 + 1.78 * YAP score -11.34
- Secondary boys: YAP % MVPA = 13.72 + 7.31 * YAP score
- Secondary girls: YAP % MVPA = 13.72 + 7.31 * YAP score -11.34

Out-of-school MVPA calibration model

n=196	ß	SE	p
Intercept (Primary level)	12.75	5.01	.012
Intercept (Secondary level)	2.61	3.41	.45
Sex	-1.61	1.95	.41
YAP x Primary level	2.01	2.06	.33
YAP x Secondary level	4.44	1.18	<.001

Calibration equations

- Primary boys: YAP % MVPA = 12.75 + 2.01* YAP score
- Primary girls: YAP % MVPA = 12.75 + 2.01* YAP score -1.61
- Secondary boys: YAP % MVPA = 2.61 + 4.44 * YAP score
- Secondary girls: YAP % MVPA = 2.61 + 4.44 * YAP score -1.61

Weekend MVPA calibration model

n=187	ß	SE	p
Intercept (Primary level)	15.17	5.02	.003
Intercept (Secondary level)	8.44	3.18	.009
Sex	-4.33	1.56	.006
YAP x Primary level	1.13	1.22	.36
YAP x Secondary level	1.44	0.90	.11

Calibration equations

- Primary boys: YAP % MVPA = 15.17 + 1.13* YAP score
- Primary girls: YAP % MVPA = 15.17 + 1.13* YAP score -4.33
- Secondary boys: YAP % MVPA = 8.44 + 1.44 * YAP score
- Secondary girls: YAP % MVPA = 8.44 + 1.44 * YAP score -4.33

Out-of-school SB calibration model

n=196	ß	SE	p
Intercept (Primary level)	34.92	12.71	.007
Intercept (Secondary level)	48.95	8.91	<.001
Sex	1.61	2.99	.59
YAP x Primary level	-0.84	5.22	.87
YAP x Secondary level	5.65	2.54	.027

Calibration equations

- Primary boys: YAP % SB = 34.92 – 0.84* YAP score
- Primary girls: YAP % SB = 34.92 – 0.84* YAP score + 1.61
- Secondary boys: YAP % SB = 48.95 + 5.65 * YAP score
- Secondary girls: YAP % SB = 48.95 + 5.65 * YAP score + 1.61
