# Supplementary material for: Calibration and Validation of the Youth Activity Profile as a Physical Activity and Sedentary Behaviour Surveillance Tool for English Youth
Source: Int J Environ Res Public Health. 2019 Oct 2;16(19):3711. doi: 10.3390/ijerph16193711 (PMC6801945; doi:10.3390/ijerph16193711)
Supplement: Supplementary file 1 [file ijerph-16-03711-s001.zip › Supplementary file 6 REVISED. Individual-level cross-validation results minutes & percent segment time.pdf]

English YAP cross-validation results

Student-level

YAP vs SWA (% segment time; 3 CV schools)

	YAP (% time)	SWA (% time)	Correlation (r)	%MAPE
In school MVPA n=129	36.9 (10.9)	35.1 (17.8)	.72	66.0
Out school MVPA n=127	15.1 (4.0)	14.7 (8.6)	.42	76.3
Weekend MVPA n=117	12.5 (4.3)	12.7 (0.1)	.52	147.3
Out school ST n=127	55.2 (16.2)	53.8 (21.9)	.79	46.3

YAP vs SWA (min/week; 3 CV schools)

	YAP (min/week)	SWA (min/week)	Correlation (r)	%MAPE
In school MVPA n=129	261.8 (82.7)	244.4 (128.2)	.11	70.8
Out school MVPA n=127	321.1 (89.3)	290.6 (171.3)	.45	83.9
Weekend MVPA n=117	224.1 (77.4)	229.5 (163.8)	.52	199.6
Out school ST n=127	1165.7 (343.8)	1056.4 (435.4)	.80	50.6
