# Supplementary material for: Calibration and Validation of the Youth Activity Profile as a Physical Activity and Sedentary Behaviour Surveillance Tool for English Youth
Source: Int J Environ Res Public Health. 2019 Oct 2;16(19):3711. doi: 10.3390/ijerph16193711 (PMC6801945; doi:10.3390/ijerph16193711)
Supplement: Supplementary file 1 [file ijerph-16-03711-s001.zip › Supplementary file 7 REVISED. Group-level cross-validation results percent segment time.pdf]

English YAP cross-validation results

School-level

YAP vs SWA (% of segment time; 3 cross-validation schools)

	YAP (% time)	SWA (% time)	YAP-SWA bias (% time)
In-school MVPA	36.5 (9.8)	34.7 (13.4)	1.6 (5.6)
Out-of-school MVPA	15.0 (2.7)	14.5 (4.4)	0.5 (1.7)
Weekend MVPA	12.3 (4.7)	12.5 (5.4)	-0.2 (0.7)
Out-of-school SB	55.9 (19.2)	54.5 (20.8)	1.4 (1.9)

Additional sub-group analyses

School-level

PRIMARY SCHOOL: YAP vs SWA (% of segment time; 4 schools)

	YAP (% time)	SWA (% time)	YAP-SWA bias (% time)
In-school MVPA	46.5 (0.8)	45.6 (1.9)	0.9 (14.5)
Out-of-school MVPA	17.7 (0.3)	20.4 (3.4)	-2.8 (3.4)
Weekend MVPA	16.7 (0.6)	17.7 (1.1)	-1.0 (0.6)
Out-of-school SB	33.6 (0.1)	33.7 (4.6)	-0.1 (4.6)

SECONDARY SCHOOL: YAP vs SWA (% of segment time; 5 schools)

	YAP (% time)	SWA (% time)	YAP-SWA bias (% time)
In-school MVPA	32.5 (4.9)	31.7 (9.6)	0.8 (4.8)
Out-of-school MVPA	14.1 (1.6)	14.0 (2.7)	0.1 (1.8)
Weekend MVPA	10.7 (1.6)	10.9 (2.8)	-0.2 (1.6)
Out-of-school SB	67.1 (0.9)	66.0 (2.6)	1.1 (2.4)

BOYS: YAP vs SWA (% of segment time; 8 schools)

	YAP (% time)	SWA (% time)	YAP-SWA bias (% time)
In-school MVPA	46.1 (6.6)	45.5 (7.3)	0.6 (4.8)
Out-of-school MVPA	16.7 (2.1)	18.1 (3.5)	-1.3 (2.6)
Weekend MVPA	15.6 (3.2)	14.6 (3.8)	1.1 (3.0)
Out-of-school SB	49.8 (18.2)	48.9 (18.5)	0.9 (3.6)

GIRLS: YAP vs SWA (% of segment time; 8 schools)

	YAP (% time)	SWA (% time)	YAP-SWA bias (% time)
In-school MVPA	32.7 (8.1)	31.3 (10.1)	1.4 (3.6)
Out-of-school MVPA	15.3 (2.3)	16.5 (5.4)	-1.3 (3.6)
Weekend MVPA	11.8 (3.1)	13.8 (4.8)	-2.0 (2.0)
Out-of-school SB	50.9 (17.4)	51.4 (17.0)	-0.6 (3.9)
